# GIS and Remote Sensing Applications in the Assessment of Change within a Coastal Environment in the Niger Delta Region of Nigeria

**DOI:** 10.3390/ijerph2006030011

**Published:** 2006-03-31

**Authors:** Yaw A. Twumasi, Edmund C. Merem

**Affiliations:** 1 Center for Hydrology, Soil Climatology, and Remote Sensing, Department of Plant and Soil Science, P.O. Box 1208, Alabama A&M University, Normal, AL 35762, USA; 2 Department of Urban and Regional Planning, Jackson State University, Jackson, MS 39211, USA

**Keywords:** Remote sensing, GIS, Coastal Environment, Environmental Change, Niger Delta

## Abstract

In the last decades, the Niger Delta region has experienced rapid growth in population and economic activity with enormous benefits to the adjacent states and the entire Nigerian society. As the region embarks upon an unprecedented phase of economic expansion in the 21^st^ century, it faces several environmental challenges fuelled partly by the pressures caused by human activities such as oil and gas exploration, housing development, and road construction for transportation, economic development and demographic changes. This continued growth has resulted in environmental problems such as coastal wetland loss, habitat degradation, and water pollution, gas flaring, destruction of forest vegetation as well as a host of other issues. This underscores the urgent need to design new approaches for managing remote costal resources in sensitive tropical environments effectively in order to maintain a balance between coastal resource conservation and rapid economic development in developing countries for sustainability. Notwithstanding previous initiatives, there have not been any major efforts in the literature to undertake a remote sensing and GIS based assessment of the growing incidence of environmental change within coastal zone environments of the study area. This project is an attempt to fill that void in the literature by exploring the applications of GIS and remote sensing in a tropical coastal zone environment with emphasis on the environmental impacts of development in the Niger Delta region of Southern Nigeria. To deal with some of the aforementioned issues, several research questions that are of great relevance to the paper have been posed. The questions include, Have there been any changes in the coastal environment of the study area? What are the impacts of the changes? What forces are responsible for the changes? Has there been any major framework in place to deal with the changes? The prime objective of the paper is to provide a novel approach for assessing the state of coastal environments while the second objective seeks a contribution to the literature. The third objective is to provide a decision support tool for coastal resource managers in the assessment of environmental impacts of development in tropical areas. The fourth objective is to assess the extent of change in a tropical ecosystem with the latest advances in geo-spatial information technologies and methods. In terms of methodology, the paper draws from primary and census data sources analyzed with descriptive statistics, GIS techniques and remote sensing. The sections in the paper consist of a review of the major environmental effects and factors associated with the problem: initiatives and mitigation measures. The project offers some recommendations as part of the conservation strategies. In spite of concerted efforts by managers to address the problems, results revel that the study area experienced some significant changes in its coastal environments. These changes are attributed to socio-economic and environmental variables.

## Introduction

In the last few decades, the Niger Delta region has experienced rapid growth in population and economic activity with enormous benefits to the adjacent states and the entire Nigerian society. From the time vast oil deposits was discovered in commercial quantities in the Delta in 1956 to the present, the Nigerian state profited immensely from the oil fortunes of the region. Since then the area has grown to become the source of foreign exchange earning for the country. Starting from the fiscal year 1975 to the new century, oil from the Delta region has on the average accounted for more than 90 percent of Nigerian exports and about 80 % of government revenues as of December, 1981[1]. In the new century, the overall contribution of the oil sector to the national economy also grew from 84 percent in 2000 and 95 percent in 2002 to about 96.7 percent in 2003 [2].

Although oil production activity in the Delta has carved a remarkable economic landscape for the country with an enormous contribution to foreign exchange earning, however, there is a negative side. Petroleum exploration has triggered adverse environmental impacts in the Delta through incessant environmental, socioeconomic and physical disasters that accumulated over the years due to limited scrutiny and lack of assessment [1]. Such institutionalized negligence has turned the area into an ecosystem under severe stress [3]. For example, oil industries carried out oil exploration for over four decades with improper environmental impact assessment procedures. Under these circumstances, the benefits of oil and gas exploration in the region did not generate a positive multiplier effect for the inhabitants of the area. Concurring with this assertion, a United Nations Environmental Program (UNEP) report opined that: the “long term cost of not acting to prevent environmental degradation has been estimated to be about $5.1 billion US dollars per year, more than 15% of the country’s Gross Domestic Product (GDP)” [4].

Elsewhere, the United States Department of Energy (USDOE) shows that since the inception of oil and gas activities, severe environmental pollution in the Niger Delta involving uninterrupted gas flaring and oil and spillage remains rampant in the region. According to the US DOE, the area has experienced 4,000 oil spills since 1960. One of the most noticeable impacts of the various oil spills and production activities has been the loss of mangrove trees. The mangrove was once a source of both fuel-wood for the local people and habitat for the area’s biodiversity, but now it is unable to withstand the high toxicity levels of petrochemicals ravaging its habitat. The spills have had adverse effects on marine life that have become contaminated, and that in turn poses enormous risks to human health from consuming contaminated seafood [5]. Oil and gas operations have not only caused degradation to the environment within an extremely sensitive ecosystem and destroyed the traditional livelihood of the Niger Delta; but at the same time, environmental pollution has affected weather conditions, soil fertility, waterways and habitats for wildlife and plant life, and has caused acid rain and decreased agricultural yield [6].

The state of the Niger Delta’s environment has attracted national and international attention due to the enormous deposits of oil and gas, and the resultant impacts coupled with a controversial revenue sharing formula that has impoverished the communities. Because of massive exploitation of oil, the ramifications for human health, local culture, indigenous self-determination and the environment are severe. Accordingly, the economic and political benefits are given more emphasis at the expense of environmental health [7–8]. More precisely other problems consist of flooding and coastal erosion, land degradation, sedimentation, lack of community participation, and weak or non-enforceable laws and regulations [9].

While the region deals with an ecological siege that accumulated over the last century, it has continued to attract development programs and plans initiated by Global Trans National Companies (TNCs) in the oil sector as well as public and private entities in the region in different facets of the economy in the 21^st^ century. Thus as the region embarks upon an unprecedented phase of economic expansion in the 21^st^ century, it faces several environmental challenges fuelled partly by the pressures caused by human activities through oil and gas exploration, housing development, and road construction for transportation, economic development and demographic changes [3]. This continued growth which in the past has resulted in environmental problems such as coastal wetland loss, habitat degradation, and water pollution, gas flaring, destruction of forest vegetation and a host of other issues requires urgent environmental assessment anchored in the use of the latest advances in geospatial information systems such as remote sensing and geographic information systems (GIS).

The problem facing the area have been compounded by gaps in previous research and limited emphasis on the assessment of change through spatial information technologies such as remote sensing and (GIS) [10]. For example, numerous studies in the literature examining environmental risk assessment, rightly point to the need for improved understanding, of monitoring on environmental issues faced by oil and gas producing communities but without geospatial technologies. Some of the leading studies on the Niger Delta in this category include a major work by Onkwuka that identified the rate at which crude petroleum activities damage habitats for wildlife and marine creatures due to the toxicity of oil production. [11]. Assessing the presence of 16 polynuclear aromatic hydrocarbons at a fishing settlement in the Delta region, Coker found all chemicals to be present at a significant concentration [12]. Ecotoxicological assessment of polycyclic aromatic hydrocarbons on contaminated sediments carried out by Brack showed that samples taken from the Warri refinery in the Delta reached extremely toxic levels [13]. While in all these studies, the authors ably identified linkages to seepages from oil discharge terminals, as a contributing factor to ecological decline, there was no use of spatial technologies as assessment tools.

Notwithstanding previous initiatives, there has not been any major effort in the literature to undertake a remote sensing and GIS based assessment of the growing incidence of environmental change within coastal zone environments of the study area. Accordingly, the current problems in the Delta underscores the urgent need to design new approaches for managing remote costal resources in sensitive tropical environments effectively in order to maintain a balance between coastal resource conservation and rapid economic development in developing countries for sustainability. GIS technology as a tool used by geographers, archaeologists, geologists and other scientists in the social and natural sciences provide opportunities for storage, manipulation and mapping of data with a spatial reference [14]. Using remotely sensed satellite imagery and GIS modeling, an analysis of the spatial distribution of land use, soil erodibility, and surface slope information in the Delta area can be obtained [15]. This project will fill that void in the literature by exploring the applications of GIS and remote sensing in a tropical coastal zone environment with emphasis on the environmental impacts of development in the Niger Delta region of Southern Nigeria.

## The Purpose and Organization of the Research

This project explores the assessment of environmental change with the use of geographic information systems and remote sensing in a tropical coastal zone environment. Emphasis is on the environmental impacts of development in the Niger Delta region of Southern Nigeria. To deal with some of the aforementioned issues, several research questions that are of great relevance to the paper have been posed. The questions include, “Have there been any changes in the coastal environment of the study area?” “What are the impacts of the changes?” What forces are responsible for the changes? Has there been any major framework in place to deal with the changes? This paper has four objectives. The prime goal for this paper is to provide a novel approach for assessing the state of coastal environments. The second objective is to contribute to the literature. The third objective is to provide a decision support tool for coastal resource managers in the assessment of environmental impacts of development in tropical areas. The fourth objective is to assess the extent of change in a tropical ecosystem with the latest advances in geo-spatial information technologies and methods. The paper has five sections. Section one provides a description of the methods and the study area. Section two presents the results and data analysis, while section three discusses the findings and their significance to GIS and remote sensing applications in a coastal zone environment. The fourth section offers recommendations for change in the current approach to policy. The final section summarizes the importance of the study to future development activities along the Niger Delta ecosystem.

## Background and Methods

### The Study area: The Niger Delta Region

**Figure 1:**
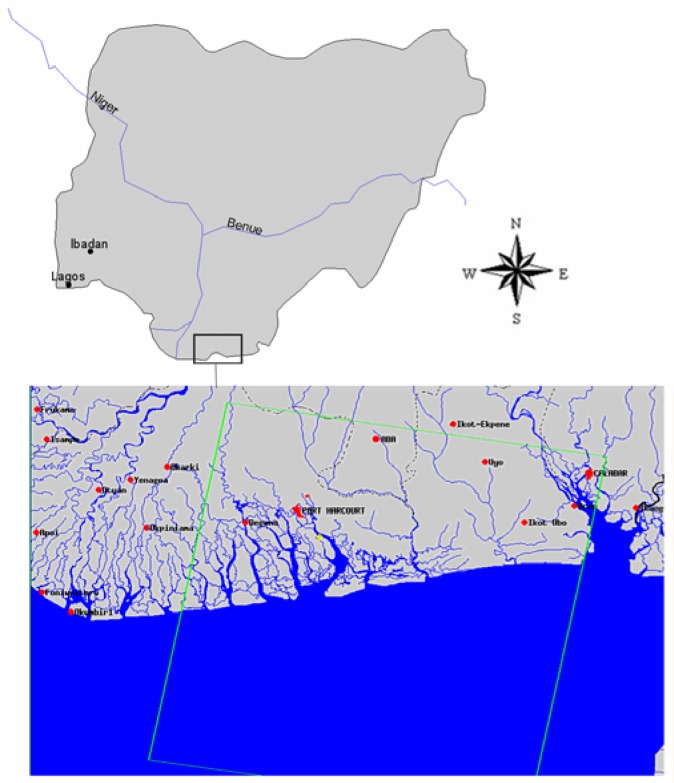
The study area. The area shows surrounding towns and cities. Insert shows the position of the study area in Nigeria

Nigeria is a former British colony and is bounded on the north by Niger, on the east by Cameroon, on the south by the Atlantic Ocean, and on the west by the Republic of Benin. The study area lies along the east of the Niger Delta (Figure 1). It is located in latitude 04° 40′ 00″N and longitude 07° 07′ 00″E. The area is home to numerous creeks and rivers. The Niger Delta region extends over an area of about 112,110 square kilometres representing about 12 percent of Nigeria’s total surface area. The region situated along a coastline of 560 km, contains about two-thirds of the entire coastline of Nigeria and nine of Nigeria’s constituent states. At the time of the 1991 census, the total population of the region stood at about 20 million, (23% of the Nigerian population). Current estimates from government sources put the total population of the region at 27 million in 2005 [16] (See Table 1).

The Niger Delta represents a unique region that possesses the world’s third largest wetland with significant biological diversity. The region has diverse mosaics of ecological types made up of five distinct ecological zones ranging from barrier island forest and coastal vegetation areas to montane habitats. The first eco-zone has the features of a mangrove forest and coastal vegetation zone with a chain of low sandy barrier islands that protects the coast of the Niger Delta, between the Benin and Imo estuaries. The second type characterized by the freshwater swamp forest zone cover approximately 17,000 square kilometers or about half of the region. The third kind mostly the lowland rain forest zone stretches over non-riverine areas flanking the Delta while the fourth zone made up of the derived savannah is found in the northern part of the region. The fifth and last eco-zone of the study area the montane zone is concentrated in the northeastern part of Cross River state of the region [16].

Part of the major concerns facing the ecosystem of the Delta as mentioned before emanates from the growing pressures mounted by development activities in the region. Because of improper management, various materials and effluents discharged into marine environment contain drill cuttings, drill mud and fluids used for stimulating oil production. Considering that the design of oil infrastructure in the area occurred without environmental considerations, during road construction, wetlands and waterways are frequently dredged and diverted to other uses at the expense of nature protection [1], [17]. In light of these problems, development activities in the study area, merits a rigorous environmental assessment with the appropriate techniques.

### Methods Used

This paper stresses a mix scale approach involving the integration of primary and secondary data provided through government sources and data bases from other organizations. The raw spatial data and satellite images used in the research came from the United States National Aeronautical and Space Administration (NASA). To answer some of the research questions pertaining to the study area, the spatial data was analyzed with descriptive statistics and remote sensing technology.

### Data Acquisition

The first step involves the identification of the variables needed to assess environmental change at regional level. The variables consist of socioeconomic and environmental information, including the amount of cropland, human settlement, water bodies, forest types, and population, (See Tables 1 and 3). The essential variables were derived from secondary sources such as government documents. This process continued with the design of data matrices for the variables covering the various periods from 1976, 1985, and 1996, 2000 and beyond. Quickened by access to databases and abstracts that are presently available, the process relied on the federal archives in Nigeria, the United States National Aeronautical and Space Administration and a host of other organizations. The spatial data acquired from NASA consists of 2 Satellite images for the separate periods of 1985 and 2000. Based upon keywords related to the term, GIS, remote sensing, coastal environment assessment, environmental change and the Niger Delta several other articles were located.

### Geo Spatial Data Processing and Analysis

Two Landsat Thematic Mapper (TM) and Enhanced Thematic Mapper Plus (ETM+) images of 4 May 1985 and 12 June 2000 were obtained for this study. The Landsat TM and ETM+ satellite data were processed using ERDAS IMAGINE 8.7 image processing software.

The images were imported into ERDAS using ERDAS native file format GEOTIFF. Since the images were in single bands, they were stacked together using ERDAS layer stack module to form a floating scene. The 1985 image was co-registered with the year 2000 image and later geo-linked to allow for the subset of both images of the study area. Enhancement of all the images using histogram equalization techniques was later performed. The images were classified using an unsupervised classification technique to identify land cover features within the study area. The remaining procedure involves spatial analysis and output (maps-tables-text) covering the study period, using ARCVIEW GIS. The spatial units of analysis consisted of the states located in the Delta region (Figure 1). Outputs for the region were mapped and compared across time. This process helped show the spatial evolution of costal environmental change in the Delta as well as changes in other variables.

## Results

This section presents the results of the data analysis by first providing a brief synthesis of the descriptive statistics and geospatial analysis (GIS and remote sensing analysis) of the assessment. Later, it highlights the factors associated with environmental change in the study area and frameworks in place to reverse the trends.

### Assessment of Environmental Change 1985–2000

**Figure 2:**
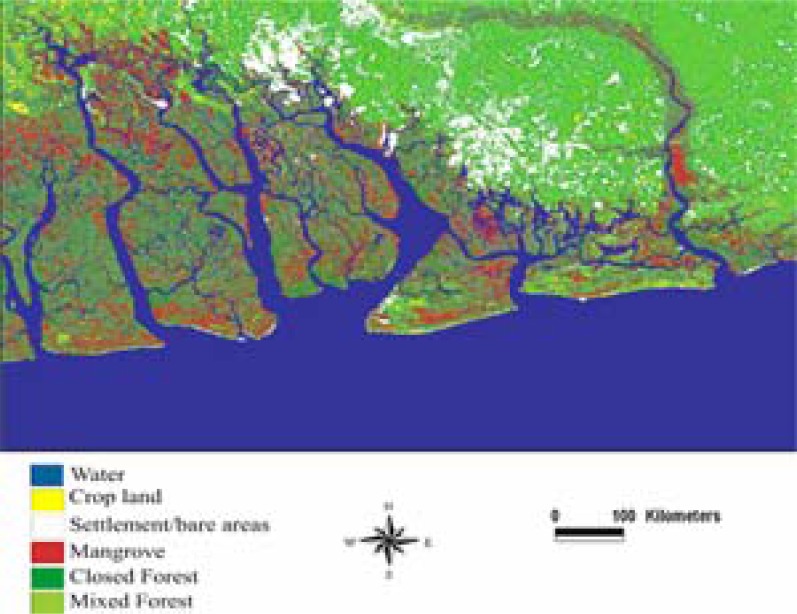
Classified image of Landsat TM, May 11, 1985

**Figure 3:**
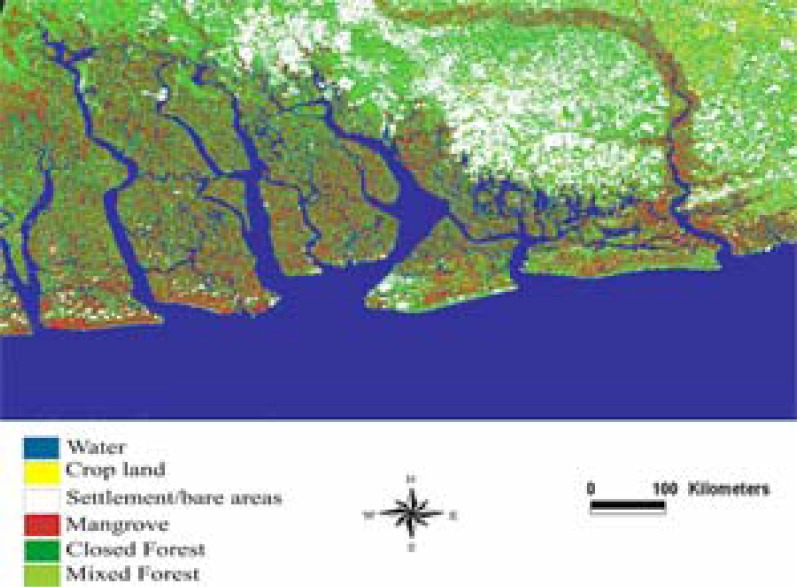
Classified image of Landsat TM, June 15, 2000

The results of 1985 and 2000 classified images presented in Table 2, Figures 2 and 3 was assessed for accuracy by comparing them to government statistics and available information from the area. From Figures 2 to 3 and Table 2, water bodies experienced a slight decline from 343,654 to 343,513 hectares. Mangrove and closed forest also posted a decline from an initial estimate of 55,410 hectares in 1985 to 37,117 hectares; and closed forest declined from 250,161 hectares in 1985 to 175,609 hectares representing an overall decrease of 33.01 and 29.80 percent respectively. While mangrove, water bodies, and closed forest were decreasing, settlement areas, cropland, and mixed forest were increasing. For instance, between 1985 and 2000, agricultural activities increased from 16,495 hectares to 23,974 hectares representing a change of 45.34 percent. Mixed forest also posted a slight change from 162,916 hectares in 1985 to 192,436 hectares in 2000, an increase of 29,520 hectares. Settlement areas had the highest increase in the region. For example, from the initial estimate of 52,738 hectares in 1985, it doubled to 108,725 hectares in 2000 representing an overall increase of 106.16 percent.

### The Frequency of Oil Spillages 1976–1996

**Figure 4:**
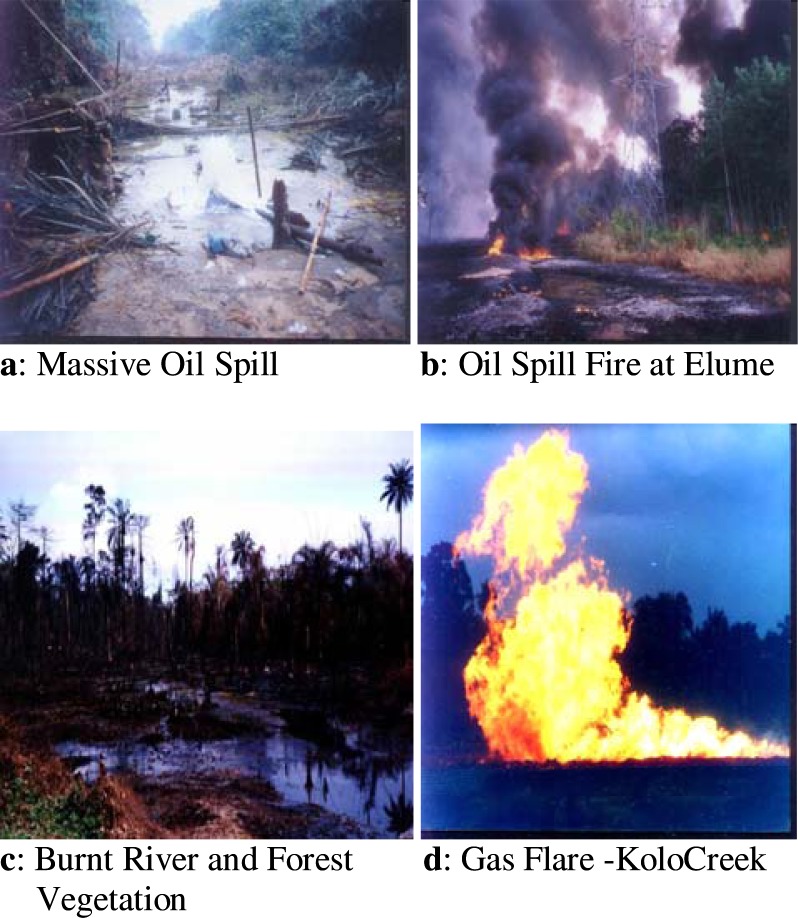
**E**xtent and nature of oil spills in selected areas of the region [27].

The frequency of oil spills from fire disasters quite rampant in the area have led to the death of thousands of community residents, contamination of water, explosions and destruction of vegetation and the freshwater ecosystem [3]. Judging from official estimates of the Nigerian National Petroleum Cooperation (NNPC), approximately 2,300 cubic meters of oil were spilled in 300 separate incidents annually. Statistics from the government indicate that in between 1976 and 1996 4,835 incidents resulted in the spillage of at least 2,446,322 barrels, of which an estimated 1,896,930 barrels were discharged to the environment. Nigeria’s biggest spill was an offshore well blow-out in 1980. This disaster resulted in the discharge of at least 200,000 barrels of oil into the Atlantic Ocean from the Texaco facility and destruction of 340 hectares of mangroves. Other spills of monumental proportions occurred in 1998 with devastating effects [8]. Historical breakdown of the spillages in the region are displayed in Table 3. From the data, the funiwa number 5 blow out of first January in 1980 resulted in the loss of 400,000 barrels to the environment. The others consists of the Oyakama oil spillage of May 1980 (30,000bbl); Oshiko oil spill in 1979 which totalled 10,000 barrels as well as the Forcados terminal oil spillage on the 6^th^ July 1979 (570,000 bbl) etc [18, 19]. See Figure 4(a–d) for visual display of the extent and nature of oil spills in selected areas of the region.

### Demographic Changes

**Figure 5:**
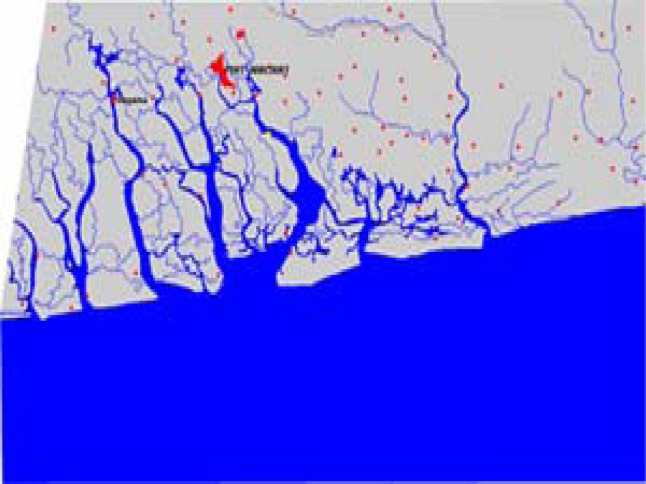
Population clusters in the study area

The last decade has witnessed the influx of people to the area in search of oil jobs and other economic opportunities causing degradation of most of the vital environmental resources. As shown in Figure 5, most of these settlements cluster around the areas experiencing highest environmental degradation. For example, the Port Harcourt area (represented with heavy red dots) contains ecologically sensitive creeks that have come under intense pollution due to the pressure mounted by human activities. During the 1991 Census, the total population for the Niger Delta region was estimated at 20 million (23% of national population). The Projections by government sources based upon yearly growth rates averaging 2.0% and 2.9%, puts the population in 2005 for the region at nearly 27 million. Nevertheless, the master plan of a baseline sample survey carried out in 2003, puts the average annual rate of population growth in most communities using household fertility and mortality data at nearly 3.1%. This implies that the population of the region today is close to 30 million. Using future projections until 2015, the region will experience a population growth between 41.5 and 48 million depending on the yearly growth rates used in computing the projections [16].

## Factors Responsible for Environmental Change and the Frameworks in Place

### Misguided Economic Policy and Over Dependency on Oil

Over the period of 1975 to date, more than 90 per cent of the nation’s exports earnings have, on average been generated from the region’s oil resources [1, 16]. Because of this dependency coupled with the strategic importance of oil to the political economy of the country in the orbit of international capital, previous governments overlooked corporate social responsibility and scrutiny of the oil sector. Accordingly, the history of the Niger Delta stands as one punctuated with decades of exogenous exploitation anchored in utter disregard for the basic rights and needs of the community. Considering that such policy is not in sync with the long term development needs of the citizens, perpetual neglect of critical human development infrastructure, including, the provision of basic social services in this area by governments and oil companies compounded the declining state of the environment facing the region [20, 21].

### Ineffective Environmental Policy and Lack of Training and Education

The first systematic surveys of the Delta’s flora and fauna conducted during the past decade showed that the forests and the animal populations of the Delta are under serious stress. The area’s most second important timber species (Abura) once common in the region has been removed by logging activities. Widespread disturbance on the Delta’s remaining forests originates from a rapidly growing Nigerian population as well as proliferation of development infrastructure that makes it easier to access the remaining areas of swamp forest. Considering the limited efforts in protected area planning in the Delta, the rapid levels of destruction epitomizes a bleak future awaiting its diverse habitats and species [3]. As in all the regulations, there is little or no enforcement. The regulatory institutions lack funding, trained staff, technical expertise, adequate in formation, analytical capability, and other prerequisites for implementing comprehensive polices and programs. Moreover, the overlapping mandates and jurisdiction between Federal Environmental Protection Agency (FEPA) and the Department of Petroleum Resources (DPR) frequently contribute to counter productive competition.

## Initiatives and Frameworks in Place

### Government Policy Efforts

The framework for oil operations in Nigeria is stipulated in the Petroleum Act, and other relevant legislations. This includes the Oil in Navigable Waters Act of 1968, the Oil Pipelines Act of 1956 as well as the Associated Gas Act of 1979, and the Petroleum (Drilling and Production) Regulations of 1969, promulgated under the Petroleum Act of 1988. Since 1988, the Federal regulations promulgated through Environmental Protection Agency (FEPA), govern environmental activities in the oil sector and other industries. The Department of Petroleum Resources (DPR) has also formulated various environmental guidelines and standards for the Petroleum Industry in Nigeria [8]. To a great extent, the content of these prescribed standards reflect those applicable to oil sectors in Europe and the United States, but surprisingly the companies still find ways to contravene the regulations [22].

### Oil Sector Initiatives

Under the existing laws, oil companies are mandated by federal laws to use precautions including the provision of state of the art technologies to prevent pollution, and act appropriately in eradicating the problems arising from petroleum (Drilling and Production Regulation, 15(2). Part of the oil sector’s legal obligations under the Environmental Impact Assessment (EIA) Act of 1992 require an EIA on the location of a proposed project that it is likely to significantly affect the environment [9]. However, the sequence of oil spill and disasters emanating from the sector casts a big doubt on the compliance readiness of oil companies [22] even though on paper the country has numerous command and control mechanisms under its policy framework for adjudicating contraventions.

### Regional Development Program

Previous policy efforts in economic development planning in the region have had a local flavour for years. This emphasis took for granted the socio economic and ecological impacts of development programs on the ecosystem of adjoining states, counties and communities where oil companies operated. Because the gravity of these problems that accumulated over the years in the Delta area, the federal government initiated a series of regional development programs to meet the challenges. The Niger Delta Development Commission (NDDC) that emerged from the process provides a platform for the focused development of the region. The NDDC’s community level process consists of three main parts: state-wide awareness and capacity building workshops to identity community problems and formulate projects for solving them, followed by community consultation and implementation, supported by a network of community research centers [23]. Under the program, communities receive protection from the menace of shoreline erosion through a series of offshore protection measures before dredging. Other aspects of the plan consist of a resettlement program for communities and the provision of free medical services to communities located within fifty kilometres of an oil flow station.

## Discussion

In spite of the frameworks in place, the results not only reveal that the study area experienced some significant changes in its coastal environments, but the region remains an ecosystem under stress. The nature and extent of this change showed some variations across time and space. The changes attributed to socioeconomic and environmental variables reflect also a host of other factors. Over all the results point to a decline in water bodies, mangrove forests, and an increase in human settlements, mixed forests, cropland and agricultural intensification as well as several cases of oil spillages which posed a major threat to the environment and natural systems of the region. Other interesting findings touch on an impending population explosion in the coastal region of the Niger Delta in Southern Nigeria. Of particular concern is the growth rate in the Niger Delta and the massive concentration of human settlements in the Port Harcourt area of Rivers state. This trend is gradually turning the Delta region into an ecological time bomb waiting to explode. This will not only threaten the carrying capacity of an already fragile ecosystem but it poses enormous challenges for both environmental and natural resource managers and policy makers in the region if not confronted with the urgency it deserves.

In light of this finding, the practical use of a mix scale approach involving primary and census data, GIS and remote sensing in tracking coastal environment change stands an update to current literature on coastal resources management in the Niger Delta of Nigeria. Considering the little efforts in the past to assess the Delta ecosystem, GIS technology as used in this paper has fulfilled a useful purpose for storage, manipulation and mapping of coastal data with a spatial reference. It also stands as an effective tool for coastal resource management. Integrated data analysis using remotely sensed satellite imagery and GIS modeling, facilitated the analysis of the spatial distribution of environmental change involving land use, land cover classification, forest and hydrology and demographic issues facing the Niger Delta environment. Such information technology is highly indispensable for the decision makers in Nigeria as they grapple with the future of development activities along the Delta ecosystem in the 21^st^ century.

## Policy Recommendations

Four recommendations for conservation strategies and environmental protection are listed below.

### Encourage Community Participation and Equity

The series of controversies surrounding the ecological decline of the Niger Delta have been partly attributed to several factors. Considering the role of policy lapses, inaction towards environmental safety of the communities and inequity, protecting the coastal environment will require active community input and equitable distribution of natural resources revenues. Thus, conserving the coastal environment of the Niger Delta demands an understanding of the socio economic needs of local communities who are constantly faced with the threat of a poisoned environment triggered by development activities. Under this setting, the authorities in Nigeria and the oil and gas industry and others should encourage the implementation of participatory approach in matters associated with oil and gas activities during the leasing process so that those communities closer to the problems can have a say on economic development matters and decisions likely to affect their local environment. This could also be achieved through meetings and training sessions on the assessment of the major environmental and social problems facing the Delta region with participation of local universities, non governmental organizations and the communities themselves, the oil sector and government agencies as well [24].

### Undertake Periodic Environmental Impact Assessment (EIA)

The development activities along the Niger Delta region has for several decades escaped a rigorous environmental scrutiny required of any fragile ecosystem in advanced nations of the world. Surprisingly, the petroleum sector of the economy undertook oil exploration without proper environmental impact assessments a situation that is untenable in advanced countries that are home to the major oil companies. To deal with these anomalies environmental impact assessment of oil and gas development projects in the region should be required before the commencement of oil and gas activities. This approach can be developed by comparing the future costs and benefits of projects on the environment before approval. The proposed model places highest priority on sets of criteria and objectives in order to gauge how the proposed activities can impact wildlife, natural habitats, hydrology, soil, wetlands and social environment. This EIA process requires also the classification of preferred alternatives to reduce identified risks to the ecosystem of the Niger Delta. While the EIA process as suggested here are frequently used in advanced nations, it is a valuable benchmark for gauging where policy interventions can be most efficiently directed given the scanty resources and support system existing in the Niger Delta [25].

### Institute an Integrated Coastal Zone Management Program (ICZM)

An integrated costal resource management approach is needed to address such a broad range of social and environmental issues facing the Delta in a sustainable manner. Integrated coastal zone management implies holistic planning and coordinating process capable of guaranteeing that large economic and social benefits from resources in the Niger Delta are not dissipated by environmentally destructive polices. ICZM represents an ecologically and socially sensitive approach to environmental management with a major divergence from the more traditional technocratic rational planning models that have proven to be out of touch and ineffective in dealing with the complexities associated with coastal problems. To accomplish its purposes ICZM builds from several actions at the national and regional level as part of an action plan to correct past environmental degradation and to modify current activities that are environmentally harmful. These include the establishment of an appropriate policy framework to support coastal resources management and environmental conservation [25]. This can be attained through a coastal management framework within the proposed Niger Delta Master Plan.

### Design a Regional Environmental Information System

Current attempts to assess the state of the environment and the environmental stewardship of economic development along the Niger Delta ecosystem are often handicapped by the lack of complete access to a comprehensive regional environmental information system. The design of a regional en vironmental information system on the ecology of the Niger Delta will serve as a decision support tool for policy makers, oil sector and research by facilitating data collection capabilities of users and access to a state of the art technical infrastructure of relevance to the management of the coastal zone. The expectation is that such an information system will help in displaying the interactions between the fragile ecosystem of the region and human activities and then sharpen the response mechanisms in dealing with the problems. It has the potential in offering a viable system for reviewing and implementing new coastal zone development projects as well as the development of effective technical infrastructure to oversee environmental monitoring of the coastal zone on a permanent basis. In this case, the paper recommends the design of a regional information system to stem the on going environmental decline of the region, as proposed by Merem [26].

## Conclusion

This project has explored the applications of GIS and remote sensing in a tropical coastal zone environment with emphasis on the environmental impacts of development in the Niger Delta region of Southern Nigeria. The paper presented a vivid overview of the issues in the literature, a review of the major environmental effects and factors associated with the problem, initiatives, and mitigation measures. Notwithstanding previous initiatives, there has not been any major effort in the literature to undertake a remote sensing and GIS based assessment of the growing incidence of environmental change within coastal zone environments of the study area. In spite of concerted efforts and initiatives to address the problems, results reveal that the study area experienced some significant changes in its coastal environments. These changes are attributed to socio-economic and environmental variables and a host of other factors.

The results point to a decline in water bodies, mangrove forests, and an increase in human settlement, mixed forests, cropland and agricultural intensification as well as several cases of oil spillages. The other interesting findings touched on the potentials for a rapid population growth in the region and the implications. This will not only threaten the carrying capacity of an already fragile ecosystem, but it poses enormous challenges for environmental and resource managers, and policy makers in the region if not confronted with urgency. To deal with these problems, the project offers some recommendations as part of the conservation strategies for the region. The recommendations consist of participatory approach, periodic assessment, coastal zone management and the design of a regional information system.

The practical use of a mix scale approach involving GIS and remote sensing tools for the assessment of environmental change provided some interesting results for coastal resources management in the Niger Delta. Moreso, it is evident that GIS technology as used by scientists for storage, manipulation and mapping of data with a spatial reference stands as an effective tool for resource management. Using remotely sensed satellite imagery and GIS modeling, quickened the analysis of the spatial distribution of environmental change involving land use, land cover classification, forest and hydrology and demographic issues facing the Niger Delta. In closing, it is our belief that successful implementation of some of the strategies could lead to effective management of the coastal environment in the Niger Delta region. Furthermore, the paper serves as an essential tool for the design of geo-spatial decision support systems for coastal resource managers in the assessment of environmental impacts of development in tropical areas. This is highly indispensable if the Niger Delta is to recover from decades of environmental decline inflicted on the area by various development actives as we move further into the opening decades of the 21^st^ century.

## Figures and Tables

**Table 1:** Population Projections High for the Niger Delta

*State*	*2005*	*2010*	*2015*	*2020*
Abia	3,230,000	3,763,000	4,383,000	5,106,000
Akwaibom	3,343,000	3,3895000	4,5537,000	5,285,000
Bayelsa	1,710,000	1,992,000	2,320,000	2,703,000
Cross River	2,736,000	3,187,000	3,712,000	4,325,000
Delta	3,594,000	4,186,000	4,877,000	5,681,000
Edo	3,018,000	3,516,000	4,096000	4,871,000
Imo	3,342,000	3,894,000	4,535,000	5,283,000
Ondo	3,025,000	3,524,000	4,105,000	4,782,000
Rivers	4,858,000	5,659,000	6,592,000	7,679,000

Total	28,856,000	33,616,000	39,157,000	45,715,000

**Table 2:** Results of the classified 1985 and 2000 images

*Classes*	*Area (ha) in 1985*	*Area (ha) in 2000*	*% Change (1985–2000)*
Water	343,654	343,513	−0.04
Crop land	16,495	23,974	45.34
Settlement/bare areas	52,738	108,725	106.16
Mangrove	55,410	37,117	−33.01
Closed forest	250,161	175,609	−29.80
Mixed forest	162,916	192,436	18.12

**Table 3:** Some Recorded Cases of Oil Spillage on the Delta from 1979–1980

*Year*	*Location*	*Category*	*Quantity in Barrel (bbl)*
1980	Funiwa number 5	blow out	400,000
1980	Oyakama	oil spill	30,000
1979	Oshiko	oil spill	10,000
1979	Forcados terminal	oil spill	570,000

Source: Ukoli 2005
